# A novel in‐frame deletion in KIF5C gene causes infantile onset epilepsy and psychomotor retardation

**DOI:** 10.1002/mco2.469

**Published:** 2024-03-23

**Authors:** Santasree Banerjee, Qiang Zhao, Bo Wang, Jiale Qin, Xin Yuan, Ziwei Lou, Weizeng Zheng, Huanguo Li, Xiaojun Wang, Xiawei Cheng, Yu Zhu, Fan Lin, Fan Yang, Junyu Xu, Anjana Munshi, Parimal Das, Yuanfeng Zhou, Kausik Mandal, Yi Wang, Muhammad Ayub, Nobutaka Hirokawa, Yongmei Xi, Guangfu Chen, Chen Li

**Affiliations:** ^1^ Department of Human Genetics and Department of Ultrasound, Women's Hospital School of Basic Medical Science Zhejiang Provincial Key Laboratory of Genetic and Developmental Disorders Zhejiang University School of Medicine Hangzhou China; ^2^ Department of Genetics College of Basic Medical Sciences Jilin University Changchun China; ^3^ Department of Genetics University of Delhi New Delhi India; ^4^ Department of Pediatrics Shenzhen Second People's Hospital The First Affiliated Hospital of Shenzhen University Health Science Center Shenzhen China; ^5^ Department of Radiology Women's Hospital Zhejiang University School of Medicine Hangzhou China; ^6^ Department of Radiology Hangzhou Hospital of Traditional Chinese Medicine Hangzhou China; ^7^ Department of Neurobiology, Department of Rehabilitation and Department of Internal Medicine of the Children's Hospital, Zhejiang University School of Medicine National Clinical Research Center for Child Health Hangzhou China; ^8^ School of Pharmacy East China University of Science and Technology Shanghai China; ^9^ Department of Cell Biology School of Basic Medical Sciences Nanjing Medical University Nanjing China; ^10^ Department of Human Genetics and Molecular Medicine Central University of Punjab Bathinda India; ^11^ Centre for Genetic Disorders Banaras Hindu University Varanasi India; ^12^ Department of Neurology and Epilepsy Center Children's Hospital of Fudan University Shanghai China; ^13^ Department of Medical Genetics Sanjay Gandhi Postgraduate Institute of Medical Sciences Lucknow Uttar Pradesh India; ^14^ Department of Psychiatry University College London London UK; ^15^ Department of Cell Biology and Anatomy Graduate School of Medicine The University of Tokyo Tokyo Japan; ^16^ Alibaba‐Zhejiang University Joint Research Center of Future Digital Healthcare Hangzhou China

**Keywords:** cargo trafficking, Drosophila model, infantile‐onset epilepsy, in‐frame deletion, Kinesin, psychomotor retardation

## Abstract

Motor proteins, encoded by Kinesin superfamily (*KIF*) genes, are critical for brain development and plasticity. Increasing studies reported *KIF*’s roles in neurodevelopmental disorders. Here, a 6 years and 3 months‐old Chinese boy with markedly symptomatic epilepsy, intellectual disability, brain atrophy, and psychomotor retardation was investigated. His parents and younger sister were phenotypically normal and had no disease‐related family history. Whole exome sequencing identified a novel heterozygous *in‐frame* deletion (c.265_267delTCA) in exon 3 of the *KIF5C* in the proband, resulting in the removal of evolutionarily highly conserved p.Ser90, located in its ATP‐binding domain. Sanger sequencing excluded the proband's parents and family members from harboring this variant. The activity of ATP hydrolysis in vitro was significantly reduced as predicted. Immunofluorescence studies showed wild‐type KIF5C was widely distributed throughout the cytoplasm, while mutant KIF5C was colocalized with microtubules. The live‐cell imaging of the cargo‐trafficking assay revealed that mutant KIF5C lost the peroxisome‐transporting ability. *Drosophila* models also confirmed p.Ser90del's essential role in nervous system development. This study emphasized the importance of the *KIF5C* gene in intracellular cargo‐transport as well as germline variants that lead to neurodevelopmental disorders and might enable clinicians for timely and accurate diagnosis and disease management in the future.

## Graphical abstract

We identified a novel heterozygous *in‐frame* deletion (c.265_267delTCA) in exon 3 of *KIF5C* in a 6 years and 3 months‐old Chinese boy with markedly symptomatic epilepsy, intellectual disability, and psychomotor retardation, resulting in the removal of evolutionarily highly conserved p.Ser90 in its ATP‐binding domain. This study emphasized the importance of the *KIF5C* in intracellular cargo‐transport as well as germline variants leading to neurodevelopmental disorders.

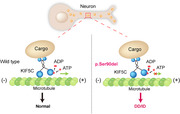

## 1 INTRODUCTION

Severe developmental delay (DD) and intellectual disability (ID), collectively referred to as cognitive impairment or mental retardation, represent an important health issue. The global incidence of DD and ID (DD/ID) among children under the age of 5 years was one in 100 live births in 2019.^1^ The clinical diagnosis of severe DD/ID in early childhood is based on a substantial delay in cognitive development. Most serious forms of DD or ID result from genetic factors.^2^ However, the specific cause of about 55–60% of patients is unknown.^1^, ^2^ Moreover, rare de novo or sporadic variants have been reported to be the most likely causes of severe DD or ID.^3^, ^4^, ^5^, ^6^, ^7^, ^8^, ^9^

Kinesin superfamily (KIF) proteins, belonging to a class of motor proteins, play a role in conveying various cargoes, such as synaptic vesicles and protein complexes, along with microtubules (MTs)^10^, ^11^, ^12^, ^13^ and also play coroles in combination with dynein and myosin superfamilies in cells. KIF proteins affect the neuronal development, plasticity, and function of axons, dendrites, and synapses by intracellular anterograde (kinesin‐mediated) and retrograde (dynein‐mediated) transport.

Studies suggested that *KIF* genes might play a crucial role in neural development in mouse models.^13^, ^14^, ^15^, ^16^, ^17^, ^18^, ^19^, ^20^, ^21^ The mice carrying homozygous knockout variants for kinesin family member 1A, 1B, 2A, 3A, 3B, 4A, 5A, and 5B (*Kif1a*, *Kif1b*, *Kif2a*, *Kif3a*, *Kif3b*, *Kif4a*, *Kif5a*, and *Kif 5b*) showed various neurological defects. The clinical symptoms mainly included abnormal changes in brain structures, a decrease in brain size, neuronal loss, reduced neuronal apoptosis rate, and deaths during pregnancy. The lethality of embryos with *Kif2a*, *Kif3a*, *Kif3b*, and *Kif5b* knockout variants highlighted the role of these genes in crucial developmental processes.^14^, ^16^, ^17^, ^22^ The conditional *Kif5a* knockout mice exhibited epileptic phenotypes.^23^ Conversely, *Kif17* overexpression in mice showed enhanced spatial learning abilities.^24^ In humans, previous studies indicated that the *KIF* genes were remarkably correlated with neurodegenerative and neurodevelopmental disorders’ pathogenesis.^25^ However, the studies, reporting the correlations of variants in *KIF* genes with DD or ID, are limited. For example, patients with ID and malformations of cortical development (MCD) were found to carry kinesin family member 2A and 5C (*KIF2A* and *KIF5C*) gene variants in 2013.^26^

The current study reported a novel heterozygous *in‐frame* deletion (c.265_267delTCA) in exon 3 of the *KIF5C* gene, which resulted in symptomatic epilepsy, seizures, ID, brain atrophy, and psychomotor retardation in a Chinese patient. This was the first *in‐frame* deletion as well as the first reported variant within the ATP‐binding domain of the KIF5C protein, which caused severe symptomatic epilepsy, seizures, ID, brain atrophy, and psychomotor retardation.

## 2 RESULTS

### 2.1 Clinical characteristics of the patient

**FIGURE 1:**
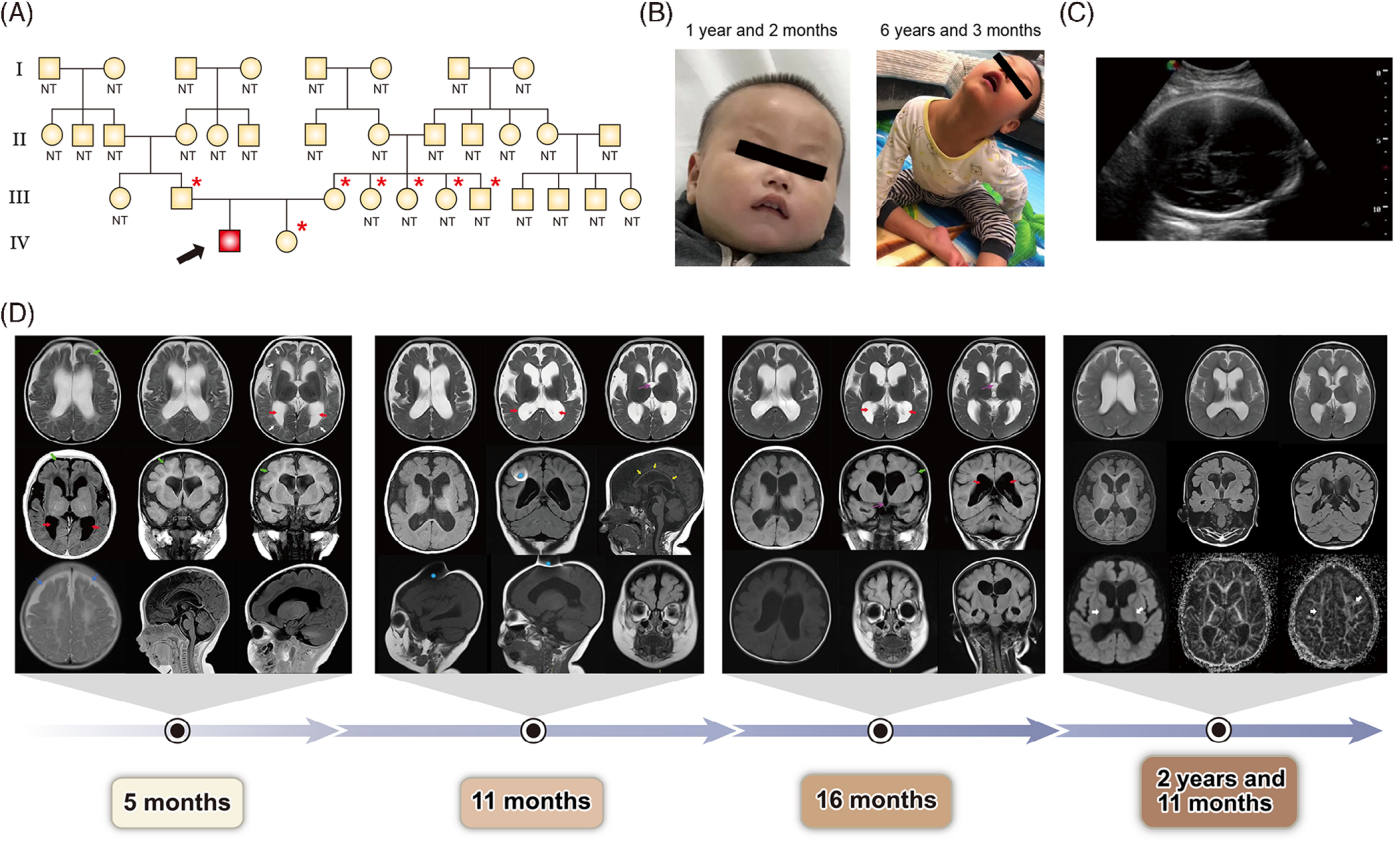
Infantile‐onset epilepsy and psychomotor retardation caused by a de novo novel homozygous in‐frame deletion in the *KIF5C* gene. (A) Pedigree of the patient family. NT, not tested using WES. Arrow denotes the proband (IV:1). Roman numerals indicate generations. Squares and circles denote males and females, respectively. (*) Symbols represent the family members confirmed using Sanger sequencing. (B) Facial phenotype of the patient. (C) Ultrasound examination of the patient at 31 weeks of gestational age revealed both lateral ventricles normal. (D) Cranial MRI results of the patient at the age of 5 months, 11 months, 16 months, and 2 years and 11 months (see Supporting Information for details). The asterisk (*) shows the MRI metal artifact.

The proband was a boy aged 6 years and 3 months; the first child of nonconsanguineous healthy Chinese parents, who had no epilepsy history in the family (Figures 1A and B). The periodic examination of the proband's mother during pregnancy revealed no abnormalities (Figure 1C). The proband was born at the 40th week of gestation via spontaneous vaginal delivery. Birth weight was 3.05 kg. Head circumference was 33 cm and birth length was 51 cm.

At the age of 5 months, the parents found that the proband was developmentally lagging his peers and was manifested with an inability to lift his head up and turn over, non‐regulated babbling and lack of social responses, and increased tone in the upper and lower limbs and toes. Deep tendon reflexes were normal and symmetrical, and the Babinski reflex was negative. Cranial MRI revealed cerebral atrophy, bilateral frontotemporal subdural effusion, and ventricular dilatation (Figure 1D). No special treatment was recommended except early intervention.

**FIGURE 2:**
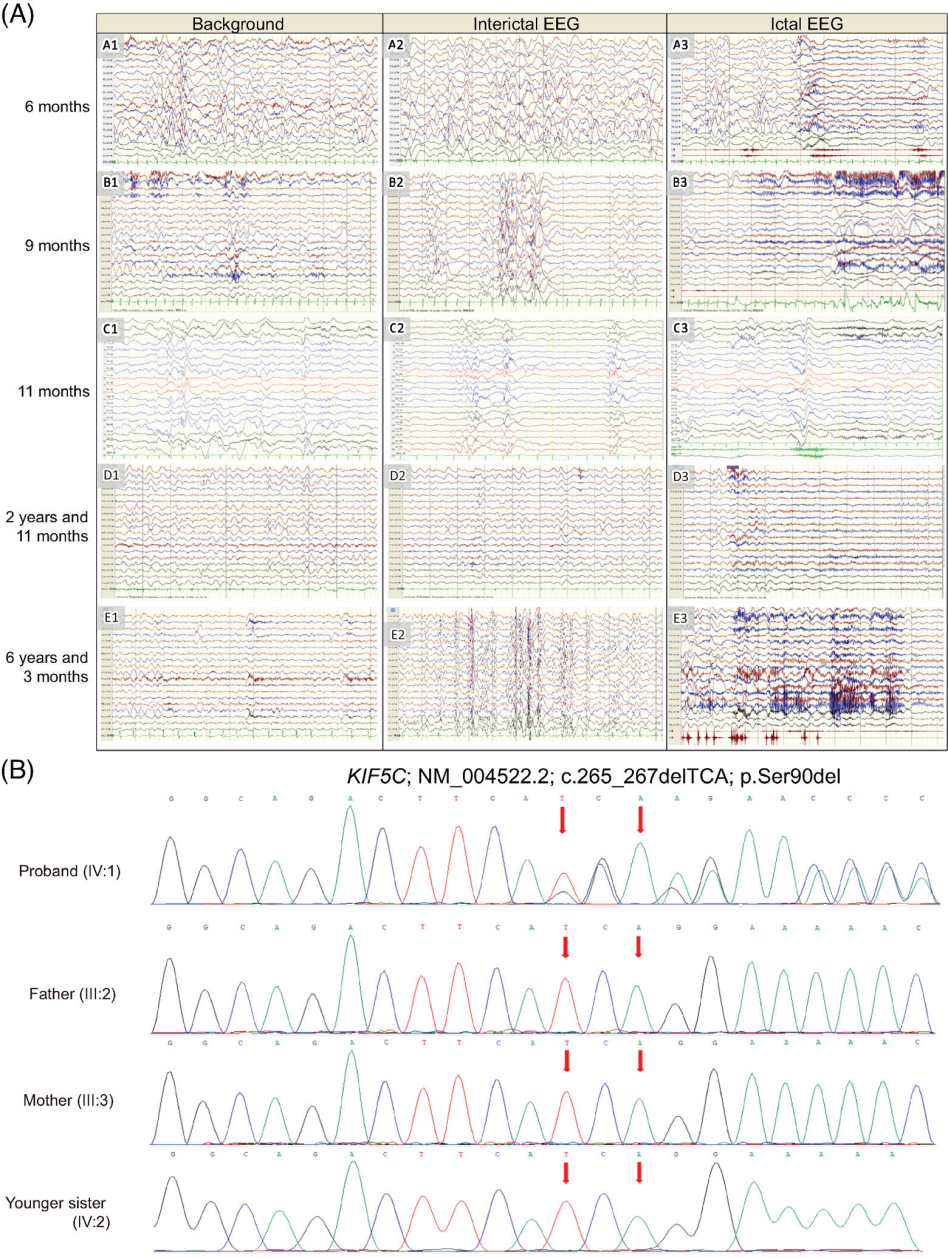
Electroencephalogram (EEG) study of the patient and Sanger sequencing. (A) EEG study of the patient (see Supporting Information for details). (B) Sanger sequencing for the patient and his family members.

At the age of 6 months, the proband was presented with daily epileptic spasms in clusters of 10−12 approximately three times a day. The re‐examination of electroencephalogram (EEG) showed hypsarrhythmia and typical epileptic spasms (Figure 2A). Based on the clinical symptoms (epileptic extensor spasms and characteristic EEG with hypsarrhythmia), the proband was clinically diagnosed with infantile spasm and psychomotor retardation. The proband was intravenously administered with vitamin B6 and without effect. In the meantime, levetiracetam and methylprednisolone were added for the treatment of seizures. The occurrences of convulsions decreased but still recurred.

Adrenocorticotropin hormone was intravenously administered till the age of 9 months; however, the effect was still modest or suboptimal. Other antiseizure medicines were also administered but discontinued due to side effects, such as repeated vomiting after adding valproate, rash after adding lamotrigine, and screaming and irritability after adding topiramate. The EEG examination at this stage was suggestive of hypsarrhythmia with isolated or series of epileptic spasms (Figure 2A).

At the age of 11 months, repeated seizures recurred. Interictal EEG showed a mass of low‐to‐medium wave amplitude with a synchronous spike‐slow wave, multi‐spike‐slow wave in the bilateral hemisphere, and simultaneous seizures (Figure 2A). MRI showed further progression of brain atrophy and ventricular dilatation (Figure 1D). The levetiracetam and clonazepam decreased the frequency of seizures to two to three times/day. The ketogenic diet was attempted at the age of 12 months and discontinued due to lack of benefit. The brain MRI showed prominent hydrocephalus at the age of 16 months (Figure 1D). The proband then gradually developed tonic and atypical absence seizures but still had spastic episodes. He was tentatively diagnosed with Lennox‐Gastaut syndrome at another hospital and orally administered with vigabatrin, clobazam, and zonisamide.

At the age of 2 years and 11 months, the EEG examination revealed no dominant rhythm in the closed occipital area, disordered sleep background, indistinguishable sleep cycles, and monitored frequent clinical seizures (Figure 2A). Cranial MRI simultaneously showed bilateral frontal megalencephaly, a remarkable reduction in white matter near the agenesis of the corpus callosum, and enlarged ventricles (Figure 1D). The diffusion tensor tractography and fractional anisotropy showed a relatively sparse bilateral fiber bundle packing. The proband was identified with myoclonic seizures, axial tonic seizures, string spasms, and clonic seizures and administered with clobazam, zonisamide, and magnesium valproate; this regimen course was continued till the writing of this article.

At the age of 6 years and 3 months, the proband was still unstable, did not follow a voice, lacked social responses, and could only sit unassisted for a few seconds supported by both his hands. The patient had increased muscle tone and involuntary limb movement. He mainly ate liquid or semiliquid food, could not speak or recognize others, and occasionally suffered from bouts of cough. Seizures were occurring daily, including tonic seizures, absence seizures, and spasms. His EEG showed bilateral occipital 6−7 Hz mixed with 4−5 Hz low‐medium amplitude *θ* rhythm, sleep stages were not easy to distinguish, and spasms or tonic seizures were monitored (Figure 2A). Chromosome microarray showed no abnormality. The proband's younger sister was 4 years old and was developing normally. Due to the lack of abnormalities in chromosomal microarray analysis, the pathogenic copy number variation and chromosomal abnormality were excluded as potential causes.

### 2.2 Identification of a de novo heterozygous deletion variant in the *KIF5C* gene

Whole exome sequencing (WES) analysis identified an *in‐frame* (c.265_267delTCA) deletion at the exon 3 of the *KIF5C* gene. This deletion variant caused the deletion of evolutionary highly conserved p.Ser90 residue of the KIF5C protein. Sanger sequencing showed that this variant was not present in his parents and family members (Figure 2B). Hence, it was a de novo variant. Furthermore, 100 normal control individuals of the same ethnicity showed no evidence of this deletion. According to the American College of Medical Genetics and Genomics (ACMG) variant interpretation guideline, c.265_267delTCA was classified as *“likely pathogenic”* variant.^27^ It was also absent in the Online Mendelian Inheritance in Man (OMIM), the Human Genome Mutation Database (HGMD), the Genome Aggregation Database (gnomAD), Leiden Open Variation Database (LOVD), and an in‐house developed database. Furthermore, neither the Exome Aggregation Consortium (ExAC), NCBI dbSNP, the ClinVar database (ClinVar), the International HapMap project (HapMap), nor 1000 Genome Database reported this variant.

In addition, there were no other potential (pathogenic or likely pathogenic) variants in other genes of the proband. However, several variants of uncertain significance,^27^ which were not considered for further studies after bioinformatics data analysis and interpretation, were found. Hence, it was confirmed that the *in‐frame* (c.265_267delTCA) deletion in the exon 3 of the *KIF5C* gene was exerting its dominant negative effect and causing disease in this proband.

### 2.3 Variant reduced the ATPase activity of KIF5C

**FIGURE 3:**
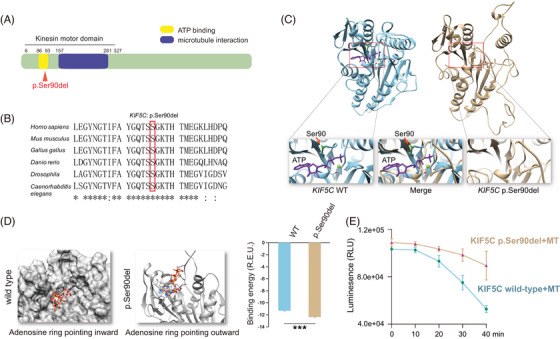
KIF5C p.Ser90del caused a reduction in ATPase activity. (A) Linear schematic overview of KIF5C, showing the variant sites. (B) Multiple sequence alignment of KIF5C nucleotide sequence centered on the KIF5C p.Ser90del patient variant showed conservation across species at this site. (C) Homology modeling of KIF5C protein using the crystal structure of KIF5B (PDB ID: 3J8Y) as a template and the p.Ser90 amino acid (in green). ATP is shown in purple. (D) Changes in the ATP binding energies between wild type and p.Ser90del variant. (E) Kinetic properties of microtubule‐dependent ATP hydrolysis by wild type and mutant KIF5C proteins (*n* = 4). MT, microtubule; *n*, technical replicates.

KIF5C acts as an intracellular motor protein, which transports vesicles and other cargo inside the cells. All previously reported variants were in the microtubule‐binding domain (MBD), while none of them were in the ATP‐binding domain (Figure 3A).^17^, ^26^, ^28^, ^29^ In addition, multiple sequence alignment showed an evolutionarily highly conserved p.Ser90, occurring across the six measured species (Figure 3B). To examine the potential effects of p.Ser90del variant on ATP binding, first, the homology models of wild type and mutant (p.Ser90del) proteins were constructed. The alignment analysis using UCSF Chimera suggested that the p.Ser90del mutant protein structure collided with the ATPase activity center (Figure 3C). This indicated that the variant might disturb the ATP‐binding domain, thereby affecting the ATPase activity of the KIF5C protein. Moreover, the changes in the ATP binding energies between the wild type and p.Ser90del KIF5C proteins were consistent with this prediction.

The molecular docking analysis of an ATP molecule into the binding pocket showed that in the wild‐type KIF5C protein, ATP could bind to the adenosine group pointing inwards into the binding pocket, while its triphosphate group was exposed outside of the pocket. In contrast, in the mutant KIF5C protein, ATP could bind to the adenosine group pointing outwards and away from the binding pocket; therefore, its triphosphate group remained buried inside the binding pocket (Figure 3D). Despite the lower binding energy of the mutant than that of the wild type, this ATP‐binding configuration might hinder the hydrolysis of ATP molecules, thereby further affecting the activity of mutant KIF5C protein.

The recombinant proteins of wild type and mutant were expressed in *Escherichia coli* and purified to validate the predicted effects of the variant on the ATPase function of the KIF5C protein. The ATP hydrolysis activity of KIF5C was then measured in vitro depending on MTs. The results showed that the ATPase activity of KIF5C was significantly reduced (Figure 3E). A previously reported p.Glu237Val variant in the MBD of KIF5C also led to the complete loss of ATP hydrolyzation.^17^ This finding was consistent with the previous in silico prediction performed using KIF5C homology models.

### 2.4 Variant altered the localization of KIF5C and the characteristics of the cell

**FIGURE 4:**
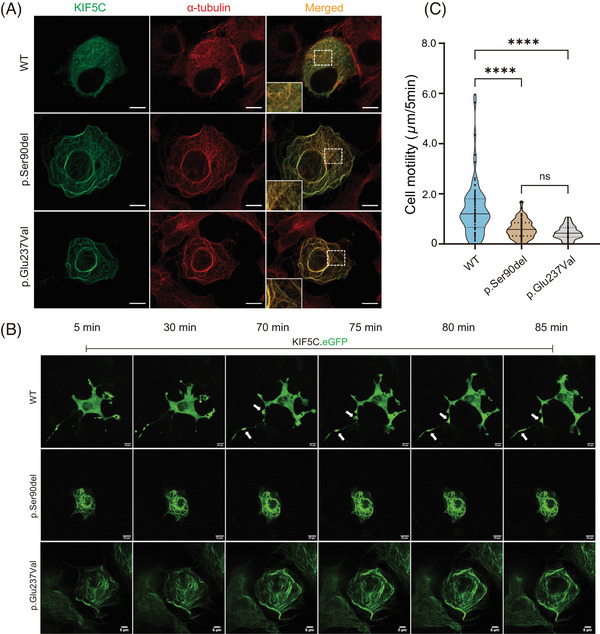
KIF5C p.Ser90del changed the protein localization and cell morphology. (A) COS7 cells expressing wild type and p.Ser90del mutant KIF5C proteins were imaged under a confocal microscope. In contrast, the p.Glu237Val mutant showed colocalization with microtubules. The scale bar represents 10 μm. (B) Live cell imaging of COS7 cells transfected wild type and p.Ser90del mutant genes. The wild type showed significant aggregation (white arrows) near pseudopodia during cell migration. Scale bars represent 10 μm (wild type and p.Ser90del) and 5 μm (p.Glu237Val), respectively. (C) Quantification of the motility of COS7 cells, expressing wild type and p.Ser90del mutant KIF5C proteins. The average length of cell migrations of each group was calculated every 5 min for 3 cells, respectively. ****: *p* < 0.0001; mean values ± SEM.

By transfecting COS7 cells with wild type and p.Ser90del constructs, the structural and functional changes upon variant were examined, and p.Glu237Val mutant was also used as a positive control (Figure 4A). The distribution and localization of KIF5C protein in the cells were observed under the microscope through immunofluorescence. The results showed that the wild‐type protein was not only colocalized with tubulin but also scattered throughout the whole COS7 cytoplasm (Figures S1 and S2). However, the p.Ser90del and p.Glu237Val KIF5C proteins did not show the distribution in the cytoplasm; instead, they showed colocalization with MTs, which indicated that these variants may affect cell morphology and migration. Therefore, live‐cell imaging was performed to investigate these effects. The results showed that the COS7 cells transfected with wild‐type KIF5C exhibited irregular cell morphology and clear pseudopodia extension, while those transfected with two mutant KIF5C plasmids (p.Ser90del and p.Glu237Val) showed regular morphologies (Figure 4B and Video S1). The significant morphological differences between the wild type and both the mutants suggested that cell migration might also be affected. Comparing the location of cells at different time points suggested that the migration ability of cells transfected with either of these two mutated KIF5C proteins was significantly weakened (Figure 4C). In addition, the wild‐type KIF5C protein showed significant aggregations near pseudopodia during cell migration.

### 2.5 Variant affected the intracellular cargo transportation

**FIGURE 5:**
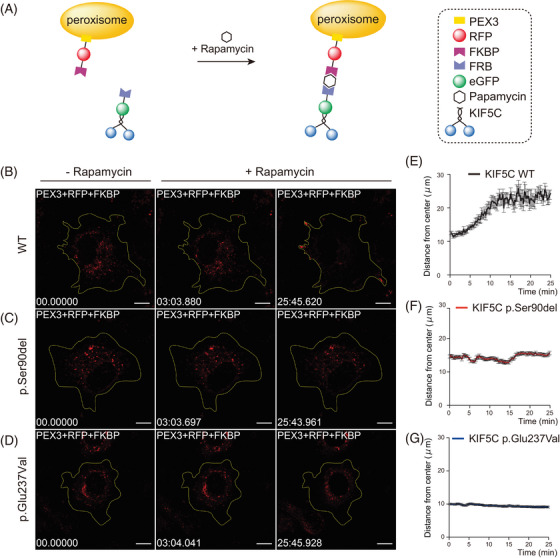
Variant in *KIF5C* gene affected cargo transport. (A) Schematic representation of the inducible cargo‐trafficking assay. (B–D) Wild‐type and mutant KIF5C–eGFP–FRB recruited PEX3–RFP–FKBP to visualize the mobility of peroxisomes in cells. The scale bar represents 10 μm. (E–G) Graph showing the peroxisome distribution in COS‐7 cells over time when the KIF5C constructs were recruited before and after the addition of rapamycin. Each value are expressed as a mean ± SEM.

The effects of the variant on the cargo‐conveying ability of KIF5C within the cells were examined using an artificial cargo trafficking assay, in which, peroxisomes were attributed to KIF5C.^30^ As an artificial cargo, the KIF5C protein was tagged at its C‐terminus with eGFP and rapamycin‐binding (FRB) domain and then expressed in COS7 cells with a peroxisome‐targeted mRFP–FKBP module (PEX3–mRFP–FKBP) (Figure 5A). The addition of rapamycin induced the dimerization of FRB and FKBP, which promoted rapid recruitment of the motor protein to the surface of the peroxisome. The results showed that peroxisomes were rapidly redistributed to the periphery of the cell upon the recruitment of wild‐type KIF5C (Figures 5B, C, and D). However, the p.Ser90del and p.Glu237Val mutant KIF5C proteins only recruited peroxisomes without transportation (Figures 5E, F, and G and Video S2).

### 2.6 p.Ser90del caused developmental defects in *Drosophila*

**FIGURE 6:**
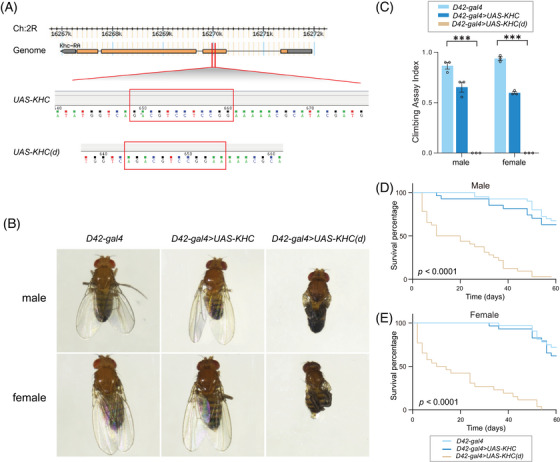
Variant caused development defects and lethality in *Drosophila*. (A) Schematic diagram of KHC and KHC(d) overexpression construction. A 95th serine (TCC) was deleted in KHC(d) line. (B) Male and female adult flies with control, KHC overexpression, and KHC(d) overexpression in motor neurons. (C) Climbing ability quantification of male adult flies with control, KHC overexpression, and KHC(d) overexpression in motor neurons. The number of male flies in the three groups was 40, 27, and 13; the number of female flies was 32, 29, and 14, respectively. (D) Life‐span quantification of male adult flies with control, KHC overexpression, and KHC(d) overexpression in motor neurons at 18°C. (E) Life‐span quantification of female adult flies with control, KHC overexpression. and KHC(d) overexpression in motor neurons at 18°C. The number of male flies in the three groups was 40, 27, and 32; the number of female flies was 32, 29, and 26, respectively.

Since the function of KIF is well‐studied in *Drosophila melanogaster*, the effects of the variant were further demonstrated in this model animal. The *Drosophila* kinesin heavy chain (dKhc) protein shares more than 70% similarities with human kinesin (based on the DRSC integrative ortholog prediction tool, DIOPT). Given the autosomal dominant role of p.Ser90del, the overexpression line of dKHC with the corresponding variant, KHC(d), was established (Figure 6A). As expected, the overexpression of KHC(d) in the *Drosophila* neural system using Elav‐Gal4 caused the death of both the male and female flies. KIF5C has been reported to be richer in motor neurons.^31^ Therefore, the function of KHC(d) was validated in motor neurons using a specific motor neuron driver, D42‐Gal4. The expression of full‐length dKHC showed no effects on the survival of adult flies, while the overexpression of KHC(d) caused visible wing phenotypes and loss of locomotion in both the male and female flies due to the long‐distance transport disorder of motor neurons caused by KHC dysfunction (Figures 6B and C). Moreover, the long‐lasting KHC(d) in motor neurons significantly affected the individual survival rate (Figures 6D and E). All the data in this study supported the conclusion that p.Ser90del of *KIF5C* might cause severe developmental arrest and affect viability.

## 3 DISCUSSION

In this study, a Chinese boy aged 6 years and 3 months with marked symptomatic epilepsy, seizures, hypsarrhythmia, ID, brain atrophy, and psychomotor retardation was investigated and analyzed. WES and Sanger sequencing found a novel heterozygous *in‐frame* deletion (c.265_267delTCA) in the exon 3 of the *KIF5C* gene.

To date, only nine studies have reported twelve cases of *KIF5C* gene variants (Table 1), including eight males and four females.^17^, ^26^, ^28^, ^29^, ^32^, ^33^, ^34^, ^35^, ^36^ Patients with the *KIF5C* variants shared seizures, significant neurodevelopmental and behavioral issues, including absence of language, and cortical abnormalities. These findings were also observed in our patient. In eight out of previous twelve cases, variants occurred at the amino acid position 237 (one was p.Glu237Val,^26^ and the other six were p.Glu237Lys),^17^, ^28^, ^29^, ^32^, ^35^, ^36^ which were all located in the MBD of the KIF5C protein. Other variant were at 268 (p.Ala268Ser) and 135 (p.Tyr135Cys).^33^, ^34^ Functional analyses for the p.Glu237Val variant revealed that the p.Glu237Val mutant heavily co‐localized with the MTs and could cause the loss of the ATP hydrolysis ability of the KIF5C protein.^26^ Investigation of the p.Glu237Lys variant effect on cellular functions using primary rat hippocampal neurons showed that this variant led to the impairment of balance between excitatory and inhibitory synapses as well as alterations in the excitability of neurons, resulting in intellectual deficit.^17^ Our novel heterozygous *in‐frame* deletion caused the removal of an evolutionarily highly‐conserved p.Ser90 amino acid, located in the ATP binding domain of the KIF5C protein. The functional data suggested that this variant also reduced the activity of ATPase, altered the KIF5C localization, cell morphology, and cell migration, affected cargo transportation, leading to a severe disease phenotype.

**TABLE 1:** An overview of previously published clinical and genetic findings of the patients with *KIF5C* variants.

Case	Gender	cDNA	Amino acid	Inheritance	Last assessment	Behavioral problems	Development	Microcephaly	Seizure	Antiseizure medication response	Cortical abnormalities	Corpus callosum anomalies	References
de Ligt et al., 2012	F	c.709G>A	pGlu237Lys	De novo	ND	ND	ND	ND	ND	ND	ND	ND	[32]
Poirier et al., 2013	M	c.710A>T	p.Glu237Val	Maternal mosaicism	1 m	ND	ND	−4 SD	1 month, clonic	CBZ	Thin cortex, polymicrogyria	Thin	[26]
	M				Medical abortion at 23GW	ND	ND	ND	–	ND	Diffuse polymicrogyria, gyral simplification	ND	
	M					ND	ND	ND	–	ND		ND	
	M					ND	ND	ND	–	ND		ND	
Jamuar et al., 2014	F	c.805G>T	p.Ala268Ser	ND	ND	ND	Developmental delay	ND	6 months, infantile spasms	ND	Posterior pachygyria, reduced white matter	Thin, dysplastic	[33]
Willemsen et al., 2014	F	c.709G>A	pGlu237Lys	De novo	ND	Stereotypic hand movements, severe automutilation	Walk with support at 9–10 y, nonverbal, severe ID	Postnatal microcephaly 49 cm (−4 SD)	6 months	ND	Frontal pachygyria	ND	[17]
Cavallin et al., 2016	M	c.709G>A	pGlu237Lys	De novo	7 y	Stereotypic hand movements, inappropriate laughter, automutilation	Independent walking at 3.5 y, nonverbal, severe DD	+0.7 SD	–	–	Frontal pachygyria, intermediate cortical thickness, nodular heterotopia	Thin, dysplastic	[28]
Al‐Shamsi et al., 2016	M	c.404A>G	p.Tyr135Cys	De novo	ND	ND	ND		ND	ND	Cortical dysplasia	ND	[34]
Michels et al., 2017	M	c.709G>A	pGlu237Lys	De novo	13 y	Stereotypic hand movements, teeth grinding, thumb sucking, rocking behavior	Wheelchair‐bound, nonverbal, severe ID	−0.8 SD	1 month, GTC	Seizure control with ASM from 3 to 12years	Disgyria in the frontal, parietal lobes and perisylvian regions	Thin	[29]
	F	c.709G>A	pGlu237Lys	De novo	11 y	Self‐injurious behavior, aggression, impulsivity	Independent walk, nonverbal, severe ID	−3.5 SD	3 months, febrile GTC	Seizure control with oxcarbazepine	Disgyria mesiofrontal pachygyria	Thin	
Duquesne et al., 2020	F	c.709G>A	pGlu237Lys	De novo	16 y	Self‐injurious behavior, aggression, impulsivity	Walk with support at 10 y, nonverbal, severe ID	Postnatal microcephaly (−5 SD)	12 months, febrile, GTC, absence	Seizure control by dual therapy (VPA, LEV)	Mild pachygyria (mesiofrontal)	ND	[35]
Naim et al., 2022	M	c.709G>A	pGlu237Lys	De novo	5 y	Mild autistic traits, Stereotypic hand movements	Unable to walk, nonverbal severe ID	Postnatal microcephaly (−4.3 SD)	1 day, GTC	Intractable (VPA, CBZ, TPM, LTG, CLB)	Frontal pachygyria	Thin	[36]
Our report	M	c.265_267delTCA	p.Ser90del	De novo	6 y	Involuntary limb movement, inattention, lacked social responses, could not speak or recognize others	Unable to walk, sit with support at 6 y, severe ID	Postnatal microcephaly 33 cm	6 months, multiple seizure types	Seizure control with CLB, ZNM, MNV from 2 to 6 years	Bilateral frontal megalencephaly, reduced white matter	Thin	

Abbreviations: ASM, antiseizure medications; CBZ, carbamazepine; CLB, clobazam; DD, developmental delay; GTC, generalized tonic‐clonic; LEV, levetiracetam; LTG, lamotrigine; MNV, magnesium valproate; ND, no data; SD, standard deviations; TPM topiramate; VPA, valproic acid; ZNM, zonisamide.

The significance of *KIF* genes, underlying DD/ID phenotypes, is less understood. Variants in the *KIF5C* gene can affect the protein function of its excitatory synapses. Recent studies showed that in the striatal and cortical neurons, KIF5 participated in regulating glutamatergic and GABAergic synaptic transmission.^37^, ^38^ The knockdown of the *KIF5C* gene could cause the impairment of contextual and spatial memory, whereas its overexpression in CA1 neurons could specifically enhance spatial memory.^39^ However, there is no specific evidence, reporting the role of the *KIF5C* gene yet. Kanai et al.^31^ reported that the *Kif5c* homozygous knockout mice were viable with reduced motor neurons to sensory neurons ratio and smaller brain size, while no gross abnormalities were found in the nervous system. The obvious explanation for this moderate phenotype might be the high structure‐function similarities among *KIF5A*, *KIF5B*, and *KIF5C* genes.^23^, ^24^

Taken together, these results strongly supported the correlations between the *KIF5C* gene and DD/ID phenotypes. The molecular mechanism, underlying the disease phenotype upon germline variant in the *KIF5C* gene, was also explored. Although *KIF5C*‐related DD/ID is extremely rare, the identification of more patients with DD/ID from different ethnicities with variants in the *KIF5C* gene might expand the variational spectrum and allow a deeper understanding of the *KIF5C* gene's role in DD/ID phenotypes. The current study showed that the *KIF5C* variant could affect intracellular cargo transportation, which might cause abnormal neuronal migration, and was supported by MCD. The variants in *KIF5C* could cause severe developmental arrest and viability in *Drosophila*. Therefore, this research strongly emphasized adding the *KIF5C* gene to the growing list of DD/ID‐associated genes.

To date, germline variants in over 400 genes have been reported to be correlated with DD/ID; however, they explain less than half of all the DD/ID cases.^40^ Moreover, patients with DD/ID usually have extreme phenotypic heterogeneity and overlapping clinical symptoms with other diseases. Therefore, WES was used in this study to identify the potentially disease‐causing variants and/or candidate genes in the patient. In the course of the exponential development in the next generation genome sequencing technologies, the clinical diagnostic potential of WES for DD/ID patients is gradually increasing. Identifying the disease‐causing variants in known DD/ID‐related genes might also provide useful clinical information, thereby allowing clinicians, patients, and their families to understand the prognoses associated with these variants. Understanding the disease etiology and identifying the genetic variants underlying the disease phenotype might allow clinicians to provide specific treatment, medication, or dietary advice. These findings also highlighted the importance of WES in providing valuable genetic information about the candidate genes and disease‐causing variants for the DD/ID patients.

In conclusion, this study dealt with a Chinese patient for 6 years, who was clinically diagnosed with infantile‐onset epilepsy and psychomotor retardation. Therefore, this study has a huge clinical significance with respect to the assessment of clinical symptoms, age at onset, disease progression, therapeutic interventions, and the application of genetic testing for accurate clinical diagnosis. To date, only two missense variants, including p.Glu237Val and p.Glu237Lys, located in the MBD of KIF5C have been reported, which cause MCD and microcephaly. This study identified the first *in‐frame* deletion variant within the ATP‐binding domain of KIF5C in the studied patient. Furthermore, a *Drosophila* disease model was established for the first time to explore the effects of the *KIF5C* gene variant on the disease pathogenicity.

## 4 MATERIALS AND METHODS

### 4.1 Case Description

The participant was a 6‐year‐old male with epileptic spasms, mental retardation and brain dysplasia. The study was approved by the Ethics Committee of Shenzhen Second People's Hospital (20180926004). When he was five months old, his chief complaints were developmental delay and referred to our hospital for early intervention training. Over the next months, he developed recurrent seizures and then treatment with anti‐seizure medications (ASMs). Genetic testing of the patient was performed as part of the diagnostic workup. The participant's parents provided informed consent prior to beginning the study.

### 4.2 Analyses of karyotype and chromosomal microarray

All the chromosomes of the proband were karyotyped using standard G‐banding to examine the structural characteristics. The copy number variations were confirmed by chromosome microarray analysis on the proband using a CytoScan HD array (Affymetrix), following the manufacturer's instructions. The analysis was performed using Chromosome Analysis Suite (v1.2.2). The threshold of the copy number was set at 10 kb with a marker count of ≥50.

### 4.3 Whole exome sequencing

The patient's genomic DNA was collected from the peripheral blood with a QIAamp DNA Blood Mini Kit (Qiagen, Hilden, Germany) following the manufacturer's protocols. The proband's genomic DNA was subjected to WES. Sequences were captured with Agilent SureSelect version 6 (Agilent Technologies, Santa Clara, CA). A high‐throughput Illumina HiSeq4000 was used to sequence the enriched library. First, the sequencing reads were aligned with GRCh37.p10 using the Burrows‐Wheeler Aligner (BWA) version 0.59. Second, the Genome Analysis Toolkit (GATK)’s Indel Realigner and Base Recalibrator were used to perform local re‐alignment and base quality re‐calibration of aligned reads, respectively. Third, single‐nucleotide variants and small insertions or deletions were identified using GATK Unified Genotyper. Finally, the annotation of variants was performed using the National Centre for Biotechnology Information Consensus Coding Sequences Database (20130630).^30^

### 4.4 Bioinformatics analysis and data interpretation

The variations in WES data with minor allele frequencies of less than 0.01 in any of the databases (the 1000 Genomes Project, HapMap, dbSNP, gnomAD, and in‐house database, containing approximately 50,000 samples of Chinese Han) were identified and selected. The annotation was performed using in silico algorithms, such as SIFT, FATHMM, Mutation Assessor, CADD, and SPIDEX, to predict the pathogenicity of variations.^41^ The variations were analyzed using the disease and phenotype databases, such as OMIM, ClinVar, HGMD, and HPO. Based on the variant interpretation guideline (2015) of ACMG, the variants were categorized as pathogenic, likely pathogenic, uncertain significance, likely benign, and benign.^27^ Further analysis of the remaining deleterious variations was performed according to the references from related published literature.

### 4.5 Sanger sequencing

Identified variants were validated using Sanger sequencing. The methods for primer designing, amplification, direct sequencing of the PCR products, and analysis of the sequencing data, have been described previously.^42^ The heterozygous novel variant was identified using WES and validated using Sanger sequencing with the primers: F: 5′‐GCCGAATCGCAATCGAAACGGGCG‐3′ and R: 5′‐GCTACTAGGAGCCGTTGGCG‐3′. The reference sequence NM_004522.2 of *KIF5C* was used.

### 4.6 Homology modeling and molecular docking

The cryo‐EM structure of the KIF5B (PDB ID: 3J8Y) was used as a template for the 3D protein modeling of wild‐type KIF5C as well as its mutant (p.Ser90del) protein. A total of 200 models were relaxed in Rosetta. The model with the lowest energy was selected for the docking of ATP with both the wild‐type and p.Ser90del KIF5C protein using the Rosetta Ligand application.^43^, ^44^, ^45^ ATP was initially placed roughly in the activity center of the protein. A total of 303 ATP conformers were produced using Frog2.^46^ During molecular docking, ligand conformations were sampled along with protein sidechain rotamers sampling. A total of 10,000 models were generated for each docking trial. After molecular docking, the top 10 models with the lowest binding energies (*interface_delta_X*) were selected as the candidates. The model with the lowest binding energy among the largest cluster of these top 10 models was used as the representative model. The visualization of all molecular dockings was performed using UCSF Chimera (v1.14).^47^ The data were analyzed using Igor Pro.

### 4.7 Plasmid constructs

The plasmids, containing the full‐length *KIF5C* sequences, were synthesized. The pcDNA3.1‐HA, pEGFP‐4T‐1, and pEGFP‐N1 plasmid (Invitrogen), containing the full length and N‐terminal 380 amino acid‐coding nucleotide sequences of *KIF5C* gene (Invitrogen), were constructed and generated from the primary plasmid. The site‐directed mutagenesis was performed using PCR with mutated primers.

### 4.8 Recombinant protein purification

The GST (glutathione‐S‐transferase)‐tagged KIF5C (KIF5C‐GST) was expressed using pGEX4T‐1 expression plasmid. The plasmids were transformed into Rosetta‐competent cells, expressed in *E. coli*, and induced by IPTG at 16°C in the shaker. Sonication was performed to lyse the cells in a lysis buffer (20 mM Tris–HCl, 500 mM NaCl, pH 7.5, 1 mM EDTA, 1% Triton‐X100, protease inhibitor cocktail from Roche). The lysed cells were centrifuged at 12,000×*g* for 20 min. Then, 0.1 mL GST‐Sepharose resin (GE Healthcare) pre‐equilibrated with 0.5 mL Thrombin protease cleavage buffer (150 mM NaCl, 20 mM Tris–HCl, pH 7.5) was added to the supernatant and rotated at 4°C for 2 hours. The beads were washed three times with Thrombin protease cleavage buffer. Then, 1U of Thrombin protease was added to the sample and incubated at 4°C overnight to cleave off KIF5C from the fusion protein.

### 4.9 Cell culture

Dulbecco's modified Eagle's medium containing 10% fetal bovine serum and 1% penicillin–streptomycin was used to culture the COS7 cell line.

### 4.10 Immunofluorescence study

The transfection of plasmids into the cells was carried out with Lipofectamine 2000 (Invitrogen). Following the fixation in 4% paraformaldehyde in PBS for 15 min at room temperature, the cells were permeabilized with 0.25% Triton X‐100 in PBS for 15 min and then blocked using blocking buffer (5% goat serum in PBS) for 1 h at room temperature. The cells were then incubated with primary antibodies anti‐KIF5C (Abcam; ab193352) and antitubulin (Sigma; T8203) diluted in block buffer at 4°C overnight. After washing with PBS four times for 5 min each, the cells were then incubated with Alexa Fluor‐conjugated respective secondary antibodies in block buffer for 1 h at room temperature.

### 4.11 Live‐cell imaging

The COS7 cells were seeded onto eight‐chamber glass coverslips, transfected with GFP‐tagged KIF5C vector using Lipofectamine 2000 (Invitrogen), and photographed under a laser scanning microscope (Zeiss LSM 800) every 5 min for 2 h using a 63X objective lens. The cell motility was plotted for six independent experiments.

### 4.12 ATPase measurements

Microtubules were purified from the pig's brain.^48^ A total of 1‐μg KIF5C protein was taken in 150‐μL kinase reaction (15 mM PIPES, pH 7.5, 10 mM MgCl_2_, 1 mM ATP) and incubated for 30 min at 30°C. The Pi contents were measured via a malachite green phosphomolybdate assay using a spectrophotometer. The ATP measurement assay was plotted for five independent experiments.

### 4.13 Inducible cargo trafficking assay

The COS7 cells were seeded on glass coverslips and transfected with plasmids KIF5C–eGFP–FRB and PEX3–RFP–FKBP using Lipofectamine 3000 (Invitrogen).^49^, ^50^, ^51^ After 16 h of transfection, the cells were washed with HBSS, and rapamycin was added at a final concentration of 400 μM. Four to five cells were selected and photographed under a laser scanning microscope (Zeiss LSM 800) every 15 s for 30–45 min using a 63X objective lens for the most acquisitions. Images were processed and analyzed using Imaris software (Oxford Instruments Andor, UK). The distance of cargo transportation was plotted for four independent experiments.

### 4.14 Drosophila stocks and breeding

Bloomington *Drosophila* Stock Center provided Elav‐Gal4 (BDSC 8765) and D42‐Gal4 (BDSC 8816). UAS‐KHC and UAS‐KHC(d) were constructed using traditional phi31‐based genome integration. The protein‐coding sequence of KHC was cloned using the specific primers as follows: KHC‐f: 5′‐gaatagggaattgggatgtccgcggaacgagagat‐3′ and KHC‐r: 5′‐aaagatcctctagaggcgtgcttgcctccatgaga‐3′. Based on the protein‐coding sequence of KHC, that of KHC(d) was cloned in two parts apart from the variant site and connected by homologous recombinase using the specific primers: KHC(d)‐f1: 5′‐gaatagggaattgggatgtccgcggaacgagagat‐3′, KHC(d)‐r1: 5′‐cgcggccgcggaattcagcttctccaccttaccct‐3′, KHC(d)‐f2: 5′‐gcatatggtcagacgtccggaaaaacgcatacgat‐3′, and KHC(d)‐r2: 5′‐aaagatcctctagaggcgtgcttgcctccatgaga‐3′. The KHC and KHC(d) coding sequences were then combined into pUAST plasmid, which was cut using *Eco*RI and *Kpn*I. The plasmids were sent to the Core Facility of Drosophila Resource and Technology (BCFly, CEMCS, CAS, Shanghai, China) to create transgenic flies.

### 4.15 Drosophila climbing assay and lifespan analysis

Climbing assays were performed as described previously with some modifications.^52^ For each genotype, adult flies were collected in an empty glass vial with a line drawn 8 cm from the bottom of the vial. After 15 min of anesthesia, all the flies were knocked down to the bottom of the tube. The files, which successfully climbed above the 8 cm mark in 10 s, were counted. Three separate and consecutive trials were carried out and the average of the results was taken. The assays were all conducted at the same time (14:00–17:00) of the day.

The lifespan of *Drosophila* was identified based on previous studies.^53^, ^54^ Flies were collected after exclusion and reared at 18°C. Every two weeks, the flies were moved to a new vial, and the percentage of surviving flies was calculated at each transfer.

### 4.16 Quantification and statistical analysis

Cell motility and cargo trafficking was performed using Imaris software (Bitplane). Statistical analysis was conducted by GraphPad Prism (GraphPad Software, Inc.). Significance of two groups or multiple groups of unmatched samples were determined using Mann–Whitney test, Kruskal–Wallis test or one‐way ANOVA with Tukey test, respectively. Survival curves were constructed using the Kaplan–Meier method and compared using the log‐rank test. All tests were two‐tailed and *p* < 0.05 was considered statistically significant.

## AUTHOR CONTRIBUTIONS

*Conceptualization of the study, project administration, patient samples, data analysis and curation, methodology, visualization, writing the original draft, review and editing of the manuscript*: Santasree Banerjee. *Data analysis and curation, methodology, visualization, writing the original draft, review and editing of the manuscript*: Qiang Zhao. *Patient samples and data collection, data analysis and curation, methodology, writing the original draft*: Bo Wang. *Conceptualization of the study, funding acquisition, patient samples, methodology, visualization, writing the original draft*: Jiale Qin. *Data analysis and curation, methodology, visualization, writing the original draft*: Xin Yuan. *Data analysis and curation, methodology, visualization, writing the original draft*: Ziwei Lou. *Patient samples, methodology, visualization, writing the original draft*: Weizeng Zheng. *Patient samples, methodology, visualization*: Huanguo Li. *Methodology, visualization*: Xiaojun Wang. *Data analysis and curation, methodology, visualization*: Xiawei Cheng. *Patient samples, methodology*: Yu Zhu. *Data analysis and curation, methodology*: Fan Lin. *Methodology, writing and editing the original draft*: Anjana Munshi. *Methodology, writing and editing the original draft*: Parimal Das. *Methodology, writing and editing the original draft*: Yuanfeng Zhou. *Methodology, writing and editing the original draft*: Kausik Mandal. *Curation, methodology, writing and editing the original draft*: Yi Wang. *Data analysis and curation, methodology, writing and editing the original draft*: Muhammad Ayub. *Conceptualization of the study, project administration, methodology*: Nobutaka Hirokawa. *Conceptualization of the study, project administration, data analysis and curation, methodology, visualization, writing the original draft, review and editing of the manuscript*: Yongmei Xi. *Conceptualization of the study, project administration, methodology*: Guangfu Chen. *Conceptualization of the study, project administration, funding acquisition, data analysis and curation, methodology, visualization, writing the original draft, review and editing of the manuscript*: Chen Li. All authors have read and approved the final manuscript.

## CONFLICT OF INTEREST STATEMENT

The authors declare no conflict of interest.

## ETHICS STATEMENT

This study was approved by the Ethics Committee of the Department of Pediatric, Shenzhen Second People's Hospital, The First Affiliated Hospital of Shenzhen University Health Science Center, and performed in accordance with the Declaration of Helsinki (Approval Number 20180926004). Written informed consent was obtained from the parents of the proband and all the participants in the family in this study.

## Supporting information

Supporting Information

**Supplementary Video S1**. Live‐cell imaging.

**Supplementary Video S2**. Cargo trafficking.

## Data Availability

The datasets used and/or analyzed during the current study are available from the corresponding author upon reasonable request.
