# Supplementary material for: A novel in‐frame deletion in KIF5C gene causes infantile onset epilepsy and psychomotor retardation
Source: MedComm (2020). 2024 Mar 23;5(4):e469. doi: 10.1002/mco2.469 (PMC10960728; doi:10.1002/mco2.469)
Supplement: Supplementary file 1 — Supporting Information [file MCO2-5-e469-s002.docx]

# SUPPORTING INFORMATION

**A novel *in-frame* deletion in *KIF5C* gene causes infantile onset epilepsy and psychomotor retardation**

Santasree Banerjee, Qiang Zhao, Bo Wang, Jiale Qin, Xin Yuan, Ziwei Lou, Weizeng Zheng, Huanguo Li, Xiaojun Wang, Xiawei Cheng, Yu Zhu, Fan Lin, Fan Yang, Junyu Xu, Anjana Munshi, Parimal Das, Yuanfeng Zhou, Kausik Mandal, Yi Wang, Muhammad Ayub, Nobutaka Hirokawa, Yongmei Xi, Guangfu Chen, Chen Li

**Supplementary Figure S1. Subcellular localization of KIF5C. A.** COS7 cells expressing wild type KIF5C proteins were imaged under a confocal microscope. The scale bar represents 5 μm.


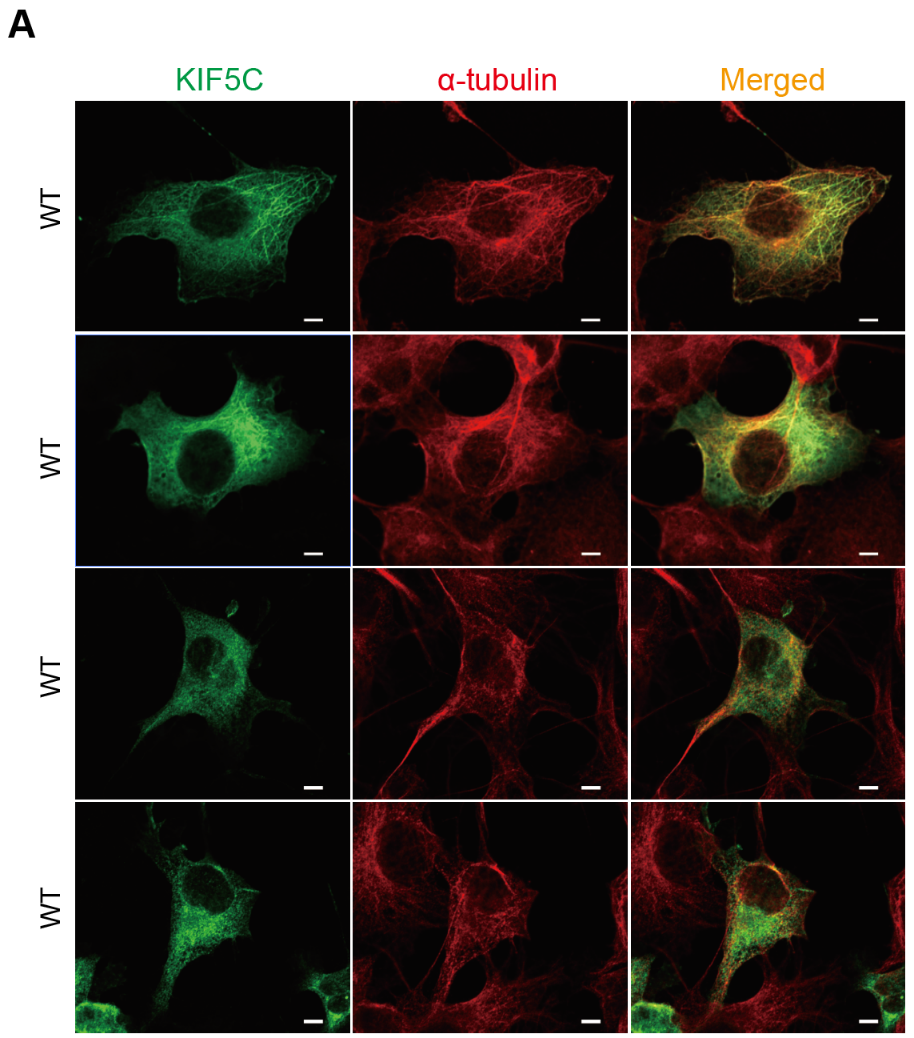


**Supplementary Figure S2. Expression of KIF5C in COS7. A.** Western blot of COS7 cells transfected with control plasmid and wild type, p.Ser90del and p.Glu237Val KIF5C vectors. Samples were separated using SDS-PAGE, transferred to Polyvinylidene Fluoride membrane, and probed with anti-KIF5C (Abcam, ab193352) and anti-tubulin (Sigma, T8203).


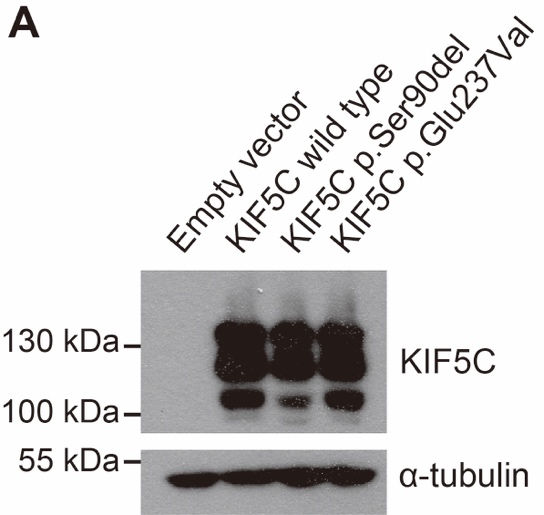


**Supplementary Information for Figure 1D.** Cranial MRI results of the patient at the age of 5 months, 11 months, 16 months, and 2 years and 11 months. (I) At the age of 5 months, an obvious dilation of the ventricles and cortical sulci in the axial T2-weighted and T1-weighted imaging (red and green arrow) and symmetrically widened extracerebral liquor spaces in the frontal and temporal regions (blue arrow) appeared. The temporal horn of the lateral ventricle was dilated as compared to the rest of the lateral ventricle. The posterior horns of the right and left lateral ventricles were 1.81 cm and 2.03 cm, respectively. The anterior horns of the right and left lateral ventricles were 1.32 cm and 1.58 cm, respectively. An irregular long T2 signal (white arrow) was observed in paraventricular white matter, which was considered to be paraventricular interstitial edema. (II) At the age of 11 months, axial images showed an enlarged bilateral ventricle at the level of the lateral ventricle. The corpus callosum was thinner in sagittal T1-weighted imaging (yellow arrow). The third ventricle protruded downward with dilation of the anterior and posterior recesses with a 9.25-mm width (purple arrow). The asterisk (*) shows the MRI metal artifact. (III) At the age of 16 months, the transverse diameter of the third ventricle was 1.08 cm (purple arrow). The degree of myelination of white matter was worse than that of normally developed infants. (IV) At the age of 2 years and 11 months, MRI showed bilateral frontotemporal lobe of pachygyria, cortical thickening, and reduced white matter (white arrow) with an unclear boundary of the gray matter, thinner corpus callosum, and supratentorial ventricular enlargement. DTT and FA showed a significant reduction in the bilateral white matter tracts with a large symmetrical distribution.

**Supplementary Information for Figure 2A. A1-A2**. Interictal EEG showed diffused slowing background activity intermingled with paroxysmal multifocal spikes or sharp waves, which were more prominent in the posterior region. **A3.** Ictal polygraphic EEG revealed diffuse voltage attenuation associated with fast activity corresponded to a burst of EMG activity and epileptic spasms, lasting for several seconds or 10 seconds. **B1-B2.** Interictal EEG showed diffuse irregular slow-wave in the background, accompanied by many multifocal spikes or mixed slow spike–waves paroxysmal, which were more pronounced in the posterior region. **B3.** Ictal EEG revealed a clinical attack period; frequent limb extension and continuous extension movements were monitored for several seconds or 10 seconds in the awakened children. EEG showed extensive low amplitude fast wave burst for several seconds to 10 seconds during the same period with or without EMG burst. **C1-C2.** Interictal EEG showed diffused slowing background activity and hypsarrhythmia with bilateral synchronous spike and poly-spike slow waves in sleep. **C3.** Ictal polygraphic EEG showed diffused high-voltage slow-wave activity with fast activity, followed by a diffused voltage attenuation associated with the transient tonia on EMG. **D1-D2.** Interictal EEG showed no dominant rhythm in the closed occipital area, disordered sleep background, and indistinguishable sleep cycles. **D3.** Ictal polygraphic EEG showed frequent clinical seizures. **E1-E2.** Interictal EEG showed posterior paroxysmal slow background activity and multifocal spikes or spike–waves, which were more prominent in the frontal regions. **E3.** Ictal EEG showed diffused low-voltage fast activity lasting for 10 seconds. The clinical manifestations were turned around, and then the upper and lower limbs straightened up for several seconds.

**Supplementary Video S1.** Live-cell imaging.

**Supplementary Video S2.** Cargo trafficking.
